# Case report: a nonfunctioning juxtaglomerular cell tumor mimicking renal cell carcinoma

**DOI:** 10.1097/MD.0000000000022057

**Published:** 2020-09-04

**Authors:** Jie Dong, Weifeng Xu, Zhigang Ji

**Affiliations:** Urology Department of Peking Union Medical Collage Hospital, Beijing, China.

**Keywords:** nonfunctioning juxtaglomerular cell tumor, renal cell carcinoma, hypertension, positron emission tomography /computed tomography

## Abstract

**Introduction:**

Based on existing literature, the juxtaglomerular cell tumor (JGCT) is a rare renal tumor, typically present with hypertension and hypokalemia. Nonfunctioning JGCT, without hypertension or hypokalemia, is extremely rare.

**Patient Concerns:**

Herein, we report a case of nonfunctioning JGCT mimicking renal cell carcinoma. The 29-year-old woman with an unremarkable past medical history presented with a left renal tumor without hypertension or hypokalemia.

**Diagnosis:**

Both CT and 18F-FDG-PET/CT suggested a malignancy, possibly renal cell carcinoma.

**Interventions:**

The tumor was then removed completely via robotic assistant laparoscopic partial nephrectomy; and pathology result was JGCT. Since the patient had no hypertension or hypokalemia, a nonfunctional JGCT was diagnosed.

**Outcomes:**

The patient recovered uneventfully, and was in good health in 6-months’ follow-up period.

**Conclusion:**

Preoperative identification of JGCT is very difficult due to the lack of specific clinical manifestations. This case teaches us that for young patients with renal tumors whose CT enhancement is not obvious at the early phase, JGCT should be considered as a differential diagnosis. Radical nephrectomy should be avoided for JGCT in consideration of its relatively good prognosis.

## 1 Introduction

Juxtaglomerular cell tumor (JGCT) is a rare disease, which is first reported by Robertson et al in 1967.^[1]^ Since then, only about 100 cases of this disease have been reported, and mostly in the form of case report. Patients with JGCT typically have severe hypertension with or without hypokalemia. However, in very rare cases, patients who have renal tumor and pathologically diagnosed as JGCT present with neither hypertension nor hypokalemia. These tumors belong to a subtype of JGCT, nonfunctioning JGCT. According to our limited knowledge, only 4 patients were diagnosis as this subtype presently.^[2–5]^ Here, we report another case of nonfunctioning JGCT, whose CT and PET/CT results might easily mislead clinicians to a diagnose of renal cell carcinoma preoperatively. Moreover, as far as we know, this is also the first case of nonfunctioning JGCT with PET/CT images which might help surgeons to further recognize this disease.

## 2 Case

**Figure 1:**
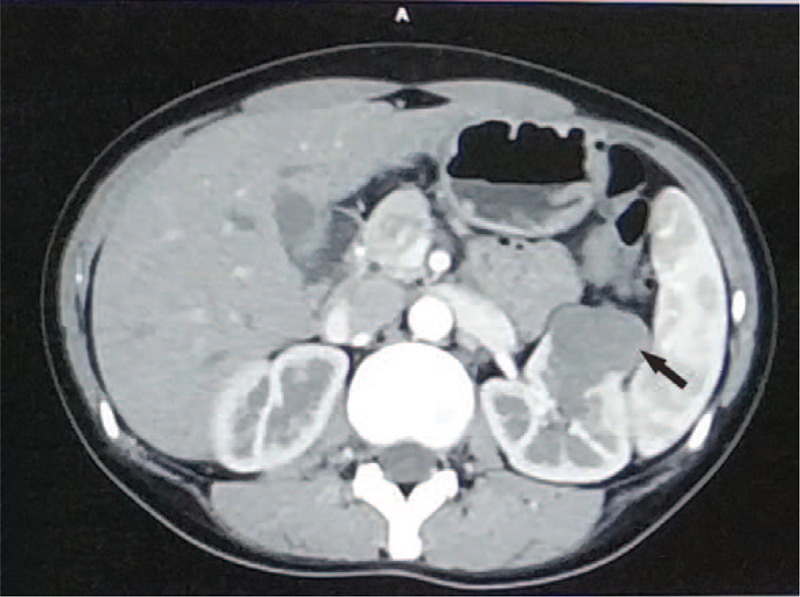
Abdominal pelvic enhanced CT. A round mass (black arrow) is showed in the middle part of the left kidney with a maximum diameter of 3.4 cm, slight enhancement in the early artery phase.

**Figure 2:**
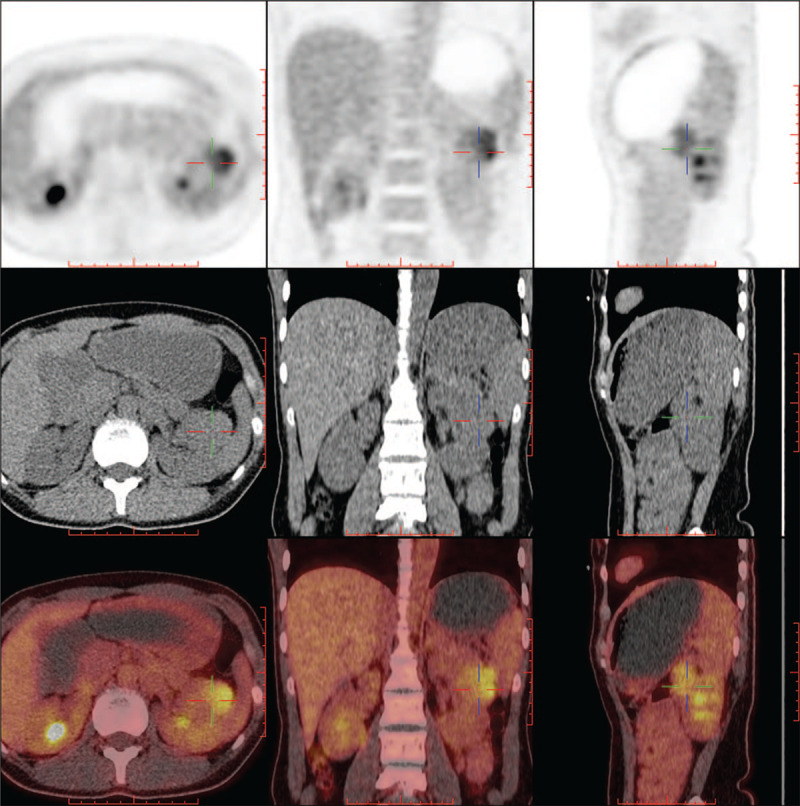
18F-FDG-PET/CT. Radioactive uptake of the tumor was abnormally increased, with a maximum standardized uptake value (SUV) of 4.6.

A 29-year-old woman was admitted to out hospital for left renal tumor which was incidentally detected by ultrasonography in a routine health examination. Ultrasound showed a low echo mass in the middle part of the left kidney, 4.3 cm in size, with clear boundary, uneven internal echo and no clear blood flow signal inside. Abdominal pelvic enhanced CT showed a round mass (black arrow) of mixed density in the middle part of the left kidney with a maximum diameter of 3.4 cm, slight enhancement in the early artery phase (Fig. 1), uneven enhancement and clear boundary in the corticomedullary phase. Renal cell carcinoma was considered for this hypovascular solid lesion. However, CT enhancement was not obvious at the early phase and it was not a typical feature of clear cell renal cell renal cell carcinoma. In order to further clarify the character of the tumor, 18F-FDG-PET/CT imaging was performed. The result showed a 4.0 × 4.2 × 6.1 cm soft tissue mass in the middle part of the left kidney, protruding forward and upward beyond the renal parenchyma. Radioactive uptake was abnormally increased, with a maximum standardized uptake value (SUV) of 4.6 (Fig. 2). It is reasonable to consider it as malignant lesion. The patient denied low back pain, hematuria, fever, frequency of urination, urgency, pain and other discomforts, and had no hypertension, diabetes and other past medical history. Personal history and family history are non-contributory. After admission, vital signs were stable, blood pressure was 130/80 mmHg, blood routine and biochemical examinations were within normal range (serum Creatinine was 53 umol/L, and Potassium was 4.1 mmol/L).

With a possible preoperative diagnosis of renal cell carcinoma, the patient underwent Robotic assistant laparoscopic partial nephrectomy. The operation was a success and the tumor was removed completely. The warm ischemia time was 10 minutes and the estimated blood loss was 50 ml. The patient recovered uneventfully from operation, and was discharged within a week after the surgery. Blood pressure and serum potassium levels were normal throughout the perioperative period.

**Figure 3:**
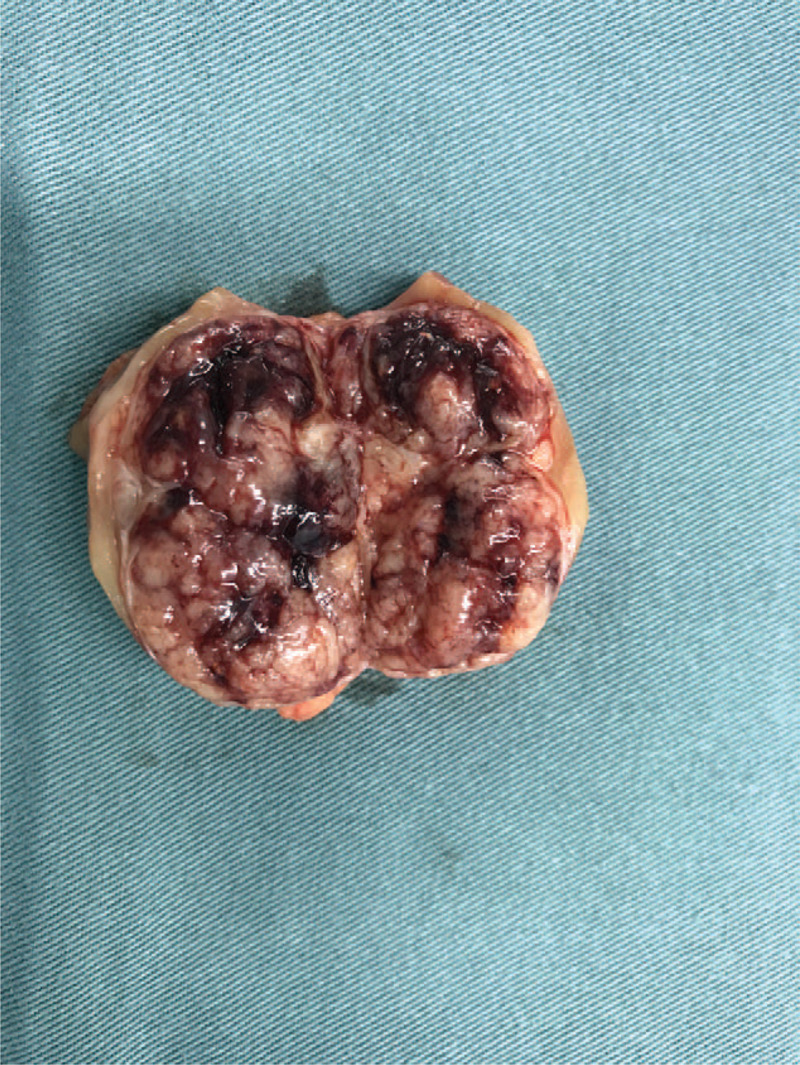
Gross section of the tumor. The 4 cm solid tumor had a complete capsule with a grayish-yellow fish-like section.

**Figure 4:**
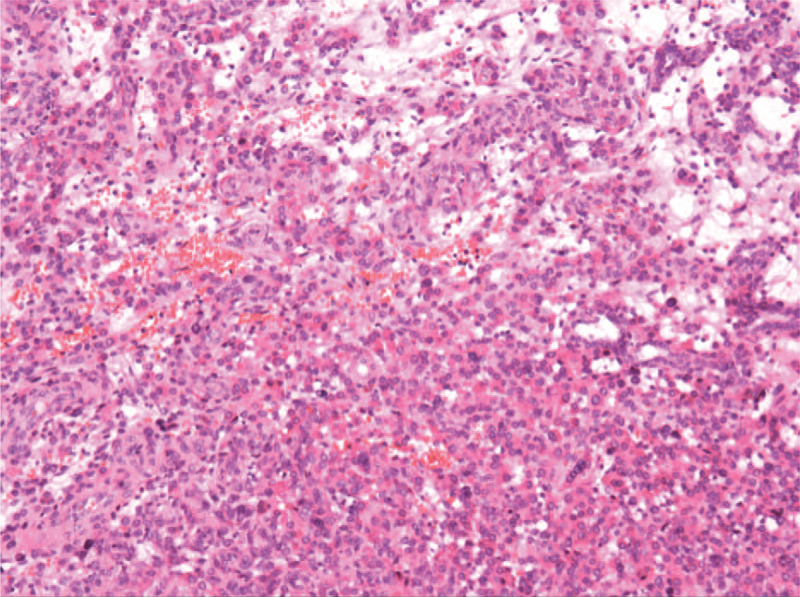
Microscopic photographs of tumors. Under light microscope, tumor cells had round or polygonal nuclei and abundant eosinophilic cytoplasm (HE ×200).

Pathological findings were vital for the final diagnosis of this patient. Grossly, the 4 cm solid tumor had a complete capsule with a grayish-yellow fish-like section (Fig. 3). Surgical margins were clear. Histologically, tumor cells had round or polygonal nuclei and abundant eosinophilic cytoplasm. An immunohistochemical study for Actin and CD3 showed strongly positive staining in the majority of tumor cells (Fig. 4). Therefore, the tumor was pathologically diagnosed as JGCT.

Since the patient had no hypertension or hypokalemia, the diagnosis of nonfunctioning JGCT in the kidney was made. The patient was followed up for 6 months and is in good health postoperatively.

## 3 Discussion

Juxtaglomerular cell tumor (JGCT) is a rare type of solid tumor of the kidney, derived from the juxtaglomerular cell. Since the tumor cells secret excessive renin,^[1]^ clinically this tumor can often cause severe hypertension and sometimes hypokalemia, in which case it would be easy to distinguish from other renal tumors (e.g., renal cell carcinoma). According to Galen et al,^[6]^ renin was first synthesized in granules as prorenin, a high molecular weight form (55,000 mol wt) and was then converted into active renin, a 44,000-mol wt form. Renin in JGCT is released by 2 pathways, mature active renin released from the granules storage and the renin precursor released immediately. However, not every patient of JGCT would suffer from hypertension,^[2–5]^ which might make the preoperative differential diagnosis rather difficult.^[5]^ The mechanism why these renin secreting tumors do not lead to hypertension remains unknown.

Nonfunctioning JGCT is mostly a solid mass of kidney found accidentally in physical examination. The patient has no special discomfort, no specific clinical manifestation such as hypertension or hypokalemia. Since most of patients with renal cell carcinoma have no clinical manifestations, and most of them are found by chance in physical examination,^[7]^ which is very similar to nonfunctioning JGCT. Therefore, it is very easy to be confused with malignant renal tumors. PET/CT scans are widely used to differentiate benign and malignant tumors, including head-and-neck, lung, cervical, and renal tumors.^[8]^ However, according to this case, which is also believed to be the first case of nonfunctioning JGCT receiving a PET/CT scan, PET/CT is not a good test to distinguish nonfunctioning JGCT (a benign tumor) from renal cancers. Also, when PET/CT was given to patients with functional JGCT, their SUV value overlaps with that of renal cell carcinoma,^[8,9]^ indicating that 18F-FDG-PET/CT cannot be used to differentiate JGCT from renal cell carcinoma. Since PET/CT could not be utilized to differentiate between renal cell carcinomas and JGCT (either functioning or nonfunctioning), clinicians have tried to explore other indicators which might help. First, Kang et al^[10]^ pointed out that age could be a useful indicator: comparing JGCT and renal cell carcinoma (RCC), the probability of developing JGCT in the 14 to 30 years’ age group was higher than that of developing RCC (*P* = .0000). While for advancing age, the probability of developing JGCT became lower than that of developing RCC. Second, enhanced CT scan might be another tool for preoperative differential diagnosis between JGCT and RCC^[11]^: the majority of renal cell carcinomas are intensely stained during the early phase (1 minute) with significant washout during the late phase (5 minutes), while JGCT rarely stained at the early phase, but stained moderately during the late phase. Therefore, age and CT findings can be used to differentiate JGCT from RCC. These indicators are especially useful for nonfunctioning JGCT because signs and symptoms related to hypertension and hypokalemia might be stronger indicators for functioning JGCT.

Although previous literatures have suggested an extremely low incidence of nonfunctioning JGCT (less than 5% of all JGCT),^[2–5]^ we speculated that the number of patients with nonfunctioning JGCT might be underestimated due to the lack of symptoms of these patients. With ultrasound becoming an increasingly widely used screening test even for healthy individuals, we might expect more nonfunctioning JGCT cases. For example, 15 cases of JGCT reported by our hospital in the recent years include 2 cases of nonfunctioning JGCT, with a much higher incidence rate compared with previous literatures. As a result, urologists should think about nonfunctioning JGCT as a potential diagnosis for asymptomatic patients with renal mass. Once JGCT is considered, nephron-sparing surgery is recommended,^[12]^ as the tumors are benign and usually small, and nearly no recurrence or metastasis.^[13]^

To sum up, nonfunctioning JGCT can occur at any age, mostly in younger adult patients. Most of the cases were found by physical examination without special clinical manifestations. This disease could not be easily differentiated preoperatively with malignant renal tumors; even PET/CT is not a good option for differentiation. Some features, such as age and enhanced CT scan, might be of some use for distinguishing this disease from renal cell carcinoma. The final diagnosis relies totally on pathological examinations. Be aware of the relatively benign course of this disease, partial nephrectomy (not radical nephrectomy) should be considered as an effective treatment.

Patient has provided informed consent for publication of the case

## Author contributions

**Conceptualization:** Jie Dong.

**Data collection:** Weifeng Xu.

**Writing – original draft:** Jie Dong.

**Writing – review & editing:** Weifeng Xu, Zhigang Ji.
